# Time-Resolved Macromolecular Crystallography at Pulsed X-ray Sources

**DOI:** 10.3390/ijms20061401

**Published:** 2019-03-20

**Authors:** Marius Schmidt

**Affiliations:** Physics Department, University of Wisconsin-Milwaukee, Milwaukee, WI 53211, USA; m-schmidt@uwm.edu; Tel.: +1-414-229-4338

**Keywords:** time-resolved crystallography, serial femtosecond crystallography, Monte Carlo integration, beta-lactamase, bacterial phytochromes

## Abstract

The focus of structural biology is shifting from the determination of static structures to the investigation of dynamical aspects of macromolecular function. With time-resolved macromolecular crystallography (TRX), intermediates that form and decay during the macromolecular reaction can be investigated, as well as their reaction dynamics. Time-resolved crystallographic methods were initially developed at synchrotrons. However, about a decade ago, extremely brilliant, femtosecond-pulsed X-ray sources, the free electron lasers for hard X-rays, became available to a wider community. TRX is now possible with femtosecond temporal resolution. This review provides an overview of methodological aspects of TRX, and at the same time, aims to outline the frontiers of this method at modern pulsed X-ray sources.

## 1. Introduction

Time-resolved macromolecular crystallography (TRX) unifies structure determination with reaction dynamics. Structures that evolve along the reaction coordinate can be determined with near atomic resolution in concert with the kinetic mechanism using the same set of crystallographic data. In favorable cases, no other information is required. Ever since the first protein structure was determined, it has been the goal of structural biologists to observe how biological macromolecules perform their function in real time. Among their many functions, which range from ligand binding to signal transduction, catalytic activity is most important. TRX has come a long way from early approaches at second-generation synchrotron light sources in the 1980s [1,2,3] to experiments at fourth generation X-ray sources that feature femtosecond (fs)-duration and extremely brilliant X-ray pulses [4,5,6]. At synchrotron light sources, the polychromatic Laue method [7] is mostly used. The broad bandwidth of the ‘pink’ X-rays allows the instantaneous collection of integrated reflection intensities. This is required as with the traditional rotation method [8], the time required to rotate the reflections through the Ewald sphere is too long to provide the required fast time-resolution. Complex software solutions needed to be developed to process the complicated, weak, and spatially overlapping macromolecular Laue data [9,10]. Lately, free electron lasers for hard X-rays (XFELs) have become available, which has propelled TRX to a new level. The enormous X-ray pulse intensities at these machines makes it possible to interrogate a tiny crystal with a single X-ray pulse that lasts only femtoseconds. This opens the previously unreachable ultrafast time scales to crystallography. As the XFEL pulses are quasi-monochromatic, new approaches to recover the integrated X-ray intensities from highly partial X-ray data were required. The integrated intensities are assembled from hundreds to thousands of observations for each individual reflection gathered from a large number (millions) of diffraction patterns. These patterns were collected from trillions of tiny crystals that were injected in serial fashion into the X-ray beam. This approach, called Monte-Carlo integration [11,12], enabled the collection of complete datasets of highly accurate reflection intensities suitable for de novo X-ray structure determination [13] and TRX experiments.

## 2. Synchrotron Light Sources

Synchrotron X-ray sources feature sub-ns X-ray pulses (around 100 ps) that are well suited for TRX experiments. Undulator sources produce intense, hard X-ray radiation which can be tuned to around 12 keV (λ~1 Å). Near atomic resolution with crystals much smaller than 50 µm is feasible even at room temperatures [14]. Undulator radiation typically carries a bandwidth of a few percent (2–5%) between 200 eV and 500 eV. At third-generation synchrotrons such as the Advanced Photon Source (APS) at the Argonne National Laboratory in Lemont, IL, the number of photons per 100-ps pulse is on the order of 5 × 10^10^ [15] in a 5% bandwidth. For exploiting this type of X-ray radiation, the Laue method [7] is employed. The advantage is that a substantial number of reflections (2–10% of reciprocal space) are excited simultaneously, and the integrated reflection intensity is collected (almost) instantaneously [16]. Since Laue reflection intensities can be scaled together by making use of the known spectral form of the incident X-ray radiation, only a few diffraction patterns, on the order of 50, are required for a complete dataset. Once the pink beam is monochromatized to typical values of about 0.01% bandwidth, (i) the overwhelming majority (>99%) of X-ray photons are disregarded and (ii) largely partial reflections are collected. Reflection intensities cannot be scaled together unless the crystal is rotated to collect the integrated reflection intensity. When the time resolution is pushed to the single-pulse limit, the crystal is effectively fixed during the pulse duration; rotations and translations are frozen in space. Each diffraction pattern is either a Laue pattern or a monochromatic still, depending on the X-ray bandwidth employed. These are some of the constraints under which a TRX experiment must be designed. Complete datasets at synchrotrons consists of a small number of diffraction patterns, each produced from multiple X-ray pulses. They require relatively large crystals and multiple crystal settings. These requirements make a TRX experiment very tricky; newest developments attempt to overcome these limitations. This review covers some of the most recent advances.

## 3. Free Electron Lasers

The free electron lasers for hard X-rays (XFELs) changed the way TRX experiments were conducted. XFELs feature femtosecond X-ray pulses with 10^12^–10^13^ hard X-ray photons (>9 keV) per pulse. These photons come within a quasi-monochromatic bandwidth of about 0.1%. Only those reflections that are lying on the Ewald sphere, and thus in reflection position, are excited by all X-ray photons available in the beam; the rest do not contribute to the diffraction pattern. The number of photons per 0.1% bandwidth (bw) in a single X-ray pulse is more than three orders of magnitude higher at XFELs (>10^12^ photons/0.1% bw) than at the synchrotron (5 × 10^10^ photons/5% bw or 10^9^ photons/0.1% bw). Accordingly, crystals smaller by three orders of magnitude might be probed by a single pulse. In addition, the laser-like properties of the XFEL allow loss-free focusing of the X-ray beam on small, nanometer-sized spots. As a result, sub-micrometer-sized crystals diffract well and produce high-resolution diffraction patterns from a single X-ray pulse [17,18]. However, after just one X-ray pulse, the acceptable X-ray dose deposited in the tiny crystal is exceeded by several order of magnitudes [19] and the crystal suffers substantial radiation damage. The ultrashort, femtosecond-long X-ray pulse ensures that the diffraction pattern is collected before the crystal is destroyed by X-ray damage [20]. The crystal is discarded and replenished by a new, pristine one. This led to the development of serial femtosecond (nano) crystallography (SFX) [17], where a large number of small crystals are injected one by one into the X-ray beam in random orientation. For this, a dense crystalline slurry is pumped through an injector nozzle device. The archetypical device is called a ‘gas dynamic virtual nozzle’ (GDVN) [21] that forms a jet of about 5 µm in diameter. The crystals move in the jet with a speed of about 10 m/s until they are interrogated by the X-ray pulse. Reflections from each microcrystal are highly partial due to the monochromaticity of the X-ray beam. Averaging over a large number of observations (typically on the order of 500 for each reflection) is required to obtain the integrated reflection intensity. This necessitates the collection of a large number (on the order of 40,000) diffraction patterns containing Bragg reflections, which need to be sorted out from a much larger number (a few million) of blank patterns that contain no reflections [22]. Since each and every microcrystal arrives at the X-ray interaction region in random orientation, each diffraction pattern needs to be indexed anew [23]. Indexing rates were initially low, but have increased to 80% and higher due to software sophistication and advances in optimizing detector geometries. The number of microcrystals required for a complete dataset also depends on the pulse repetition rate of the X-ray light source. For example, the Linac Coherent Light Source (LCLS) located at the SLAC National Accelerator Laboratory in Menlo Park, CA, features a 120-Hz repetition rate. The use of 8-ms gaps between the X-ray pulses allows a large number of microcrystals to pass by without being interrogated by X-rays. The jet volume passing by in 8 ms is about 1.6 × 10^−6^ mL, given the jet diameter of 5 µm and speed of 10 m/s mentioned above. Assuming a crystal density of 10^11^/mL, 160,000 crystals will pass by, and only one is interrogated by the X-ray pulse. Moreover, the probability that an X-ray beam of 1-μm diameter intersects a 1-μm crystal in the jet is low, on the order of 5%, even at high crystal volume densities. As a result, for a single diffraction pattern with Bragg reflections, about 3 million microcrystals are required. To obtain 50,000 patterns of which 40,000 can be indexed for a dataset, therefore, on the order of 150 billion (1.5 × 10^11^) microcrystals are needed, which is actually 1.5 mL of the dense crystalline slurry mentioned. In reality, this number is higher due to experimental challenges, and lies on the order of 5 mL, which contains about 100 mg of protein and half a trillion (0.5 × 10^12^) microcrystals.

To reduce the number of crystals required for a dataset, two strategies among many are immediately conceivable: (i) the jet speed can be reduced so that a smaller volume of dense crystalline slurry passes by, and (ii) the pulse repetition rate of the XFELs can be increased. Both strategies are successfully pursued. A device that produces a much slower jet was developed [24] that allows tightly spaced volumes to be interrogated by the low repetition rates of most XFELs. The device slowly (at nL/min speeds) extrudes a viscous material containing microcrystals. This drives crystal consumption down to less than one milligram, even at the LCLS with its 120-Hz pulse repetition. The first material, which was used to form the viscous jet, was a lipid that formed a lipidic cubic phase. The lipidic cubic phase has a viscosity comparable to toothpaste. Therefore, the injector device has been also called the ‘toothpaste’ injector. The ‘toothpaste’ injector was very successfully used for many membrane proteins, including the rhodopsins [25,26] and GPCRs [27,28]. At the Japanese XFEL, the Spring-8 Ångstrøm Compact X-ray LAser (SACLA), an injector system was developed that uses various viscous materials such as nuclear-grade grease (superlube) as jet material [29,30]. Crystals are extruded into the 30-Hz repetition rate X-ray pulses at SACLA. This method has been successfully used for soluble [31,32] and membrane protein crystals [26,33]. Other slow injectors rely on electric fields to produce a finely spun liquid jet [34].

High X-ray pulse repetition rates necessitate closely spaced electron bunches in the linear accelerator (LINAC). The displacement currents required to generate the electric fields to accelerate these bunches in the LINAC are substantial, which limit pulse repetition rates with warm copper technology. At cryogenic (liquid helium) temperatures, superconducting technology can provide repetition rates of up to MHz. The first superconducting XFEL is the European XFEL (EuXFEL) in Hamburg, Germany. The EuXFEL is designed to provide 10 pulse trains per second, where each pulse train ultimately consists of up to 2700 X-ray pulses at a repetition rate of 4.5 MHz (with 200 ns between the pulses within the train). After a gap of about 99.5 ms, the pulse train repeats. As a result, the EuXFEL will produce 27,000 pulses per second, which is more than two orders of magnitude faster than the LCLS. However, the X-ray camera that collects the Bragg patterns must operate at a MHz rate in order to separate the diffraction patterns produced by the individual pulses in the pulse trains. An appropriate camera, called the adaptive gain integrating pixel detector (AGIPD), has been developed that is capable of collecting diffraction patterns with a 4.5-MHz frame rate [35]. It has been shown that MHz data collection is possible with the AGIPD and specially designed GVDNs that produce jets with 30–100 m/s velocity [36,37]. At this velocity, subsequent pristine jet volumes are not, or only insignificantly, affected by X-ray pulses intersecting the jet further downstream. Still, there are 99.4-ms gaps between the pulse trains. As a result, about 100 million crystals per second pass by and are not interrogated by X-rays. However, if specifications are fully adhered to (27,000 X-ray pulses/s), the collection of a dataset will only take about 40 s (again assuming 40,000 indexed patterns with a hit rate of 5% and an indexing rate of 80%). Note that the hit rate is only dependent on the crystal density in the jet and does not change despite the faster X-ray repetition rate. Consequently, only 4 billion microcrystals will be required for a dataset at the EuXFEL, as opposed to 150 billion at the LCLS. This jet also produces substantial waste during the 99.4-ms breaks between the pulse trains. A ‘jet-on-demand’ that only delivers crystals during the approximately half-millisecond-long X-ray pulse trains would reduce this waste by more than two orders of magnitude. Superconducting XFELs such as the LCLS-II with constant repetition rates (from a few kHz up to 1 MHz) would certainly reduce sample consumption even more and speed up data collection enormously. As the gaps between the X-ray pulses are 10 µs at 100 kHz, a high-speed jet would not be necessary. With conventional jet velocities of about 10 m/s, 100 µm of jet material between the X-ray pulses would ensure that the next, fresh crystal is not affected by a previous X-ray pulse [36,37,38,39]. A sample reduction by a factor of 50 relative to the EuXFEL and almost a factor of 1000 relative to the LCLS operating at 120 Hz can be expected. Rather than consuming more than 100 mg of sample at the LCLS, as required today, less than 0.1 mg will be only required per dataset. At 100 kHz, the collection of a complete dataset will only take 10 s (assuming 5% hit rate and 80% indexing rate), and it should be possible to collect hundreds of datasets per hour. Since time-resolved experiments require comprehensive time-series of time-resolved X-ray data that may consist of a large number of complete datasets, high-repetition-rate XFELs such as the EuXFEL or upgrades to the LCLS are indispensable. Any low-repetition-rate method used to deliver crystals, such as the viscous (‘toothpaste’) jet, the fixed target [40], or providing drops on demand [41], cannot be used at high-repetition-rate XFELs unless they can be modified to cope with the enormous repetition rates. It is possible that droplets of liquid and/or viscous material could be injected directly into the X-ray interaction region with high repetition rates using fast and precise instrumentation. This will allow the interrogation of soluble protein and membrane protein microcrystals alike. Then, the dream of following a biomolecular reaction with closely spaced time-delays would become a reality. Methods and concepts to analyze these data were initially developed using synchrotron TRX data [42,43,44], and could be also applied to XFEL data as shown previously [5]. However, on the ultrafast time-scale an interpretation by conventional, thermal bath-driven chemical kinetics as described in textbooks [45] is not applicable. Reactions proceed on excited-state potential energy surfaces (ES-PES) and are driven by electrostatic interactions [46]. Transitions to the ground-state potential energy surface (GS-PES) may occur through conical intersections [46]. The description of the dynamics on the ultrafast time scale as well as the characterization of functional modes and structures near the conical intersection now is an important goal of TRX. In this review, TRX experiments using low-repetition-rate XFEL sources are covered. They, for the first time, allow insight into biomolecular reactions with versatility and speed that has been impossible before.

## 4. Reaction Initiation

**Figure 1:**
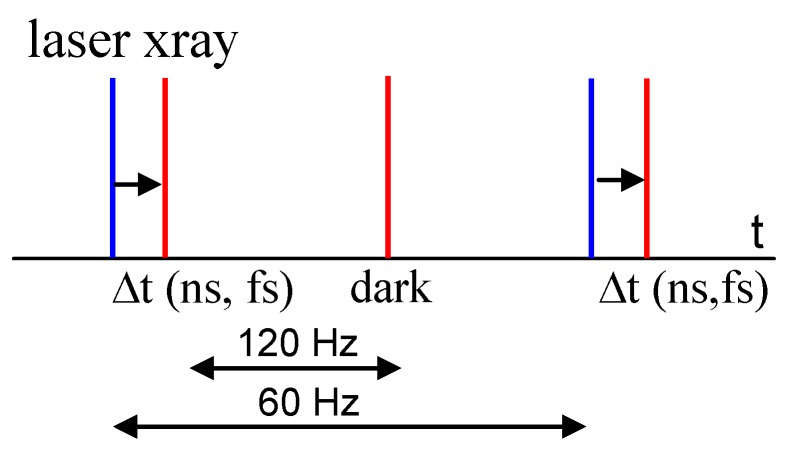
A time-resolved macromolecular crystallography (TRX) experiment with the pump–probe method. The pump laser pulse (blue bar) starts the reaction, and the ultrashort X-ray pulse (red bar) probes the progress of the reaction after a time delay, ∆t. Here, X-ray data collection in the dark as the reference is interleaved with data collection after laser light activation. The X-ray data collection rate is 120 Hz, as available at the LCLS, and the laser repetition rate is 60 Hz in this example.

Despite the larger protein consumption, macromolecular reactions can be investigated with time-resolved serial crystallography (TR-SX) at the synchrotron [47] or with TR-SFX at the XFEL [4]. In fact, the serial crystallography approach seems to be predestined for these applications. When a reaction is started in the microcrystals upstream of the X-ray interrogation zone (see Figure 1), a time-resolved dataset is collected without additional complications. The challenge is to provide a versatile system to start reactions in crystals. This is most straightforward when the reaction can be initiated by light in and near the visible range. This requires (i) the presence of a light-sensitive compound, a chromophore, and (ii) that absorption of light is functional and triggers a biologically meaningful reaction. Examples are provided further below. The chromophore is activated by an intense, short or ultrashort optical laser flash, which generates a response of the biomolecule on various time scales. The laser flash is synchronized to the accelerator radio frequency of the pulsed X-ray source. This way, the time delay ∆t between laser and X-ray pulses (Figure 1) can be selected and adjusted. When time delays are substantially shorter than the 100-ps pulse duration of the synchrotron, XFELs are required. A complication arises caused by the substantial temporal jitter of the XFEL beam which is around 300 fs [48]. If the biological reaction is to be observed on the femtosecond time scale, the temporal differences between laser and X-ray pulses must be measured on a shot-by-shot basis using a timing tool [5,6,49,50]. This enhances the time resolution substantially beyond the jitter. Another, more general method to trigger reactions in light-insensitive biomolecules and enzymes is described later on. The result of a successful TRX experiment is one or more complete datasets of reflection intensities collected after light activation and a complete reference dataset collected in the dark (Figure 1).

## 5. Data Collection and Structure Determination

During X-ray exposure, a crystal diffraction pattern is collected by the detector. Commercially available detectors installed at synchrotrons output a file format that typically consists of a header that includes information such as pixel size, pixel numbers in x and y dimensions, detector distance from the crystal, beam center, etc., followed by the raw data as a byte stream. Data analysis follows established procedure [16,51]. Detectors available at XFELs such as the CSPAD [52] or the AGIPD [35] stream the content of multiple active detector panels individually. This information needs to be gathered and stored in a user-accessible form. In addition to the detector image, additional information is collected, such as the spectral traces of the timing tool to determine the relative laser pulse to X-ray pulse delays [49,50,53]. All the data are concentrated on a per-event (exposure) basis in files based on the hierarchical data format (HDF). Diffraction patterns containing Bragg reflections are identified in the stream of detector data using a hit-finder such as the popular Cheetah software [22]. Since the diffraction patterns arise from microcrystals in random orientation, each pattern needs to be indexed anew, after which the highly partial reflection intensity can be extracted. This requires specialized software, such as CrystFEL [23,54], designed to index and analyze still exposures. After averaging a large number of observations for each reflection, a complete dataset covering 3D reciprocal space is obtained. This method of data processing has been named, based on the random nature of the crystal orientation, Monte-Carlo integration [11].

### 5.1. Twinning

With the serial approach, a complete dataset generated is composed of observations from a large number of different crystallites. This leads to an additional complication: In some space groups, some of the cell axes are identical in lengths. The indexing algorithm assigns cell axes randomly from crystallite to crystallite. A consistent indexing convention cannot be maintained. This may lead to an indexing ambiguity. Even when the cell axis lengths are not identical, such as in monoclinic space groups, some of the axes can have almost the same lengths, or there may a diagonal that by chance is of similar length compared to another cell axis. An example is sperm whale myoglobin crystallized in space group P2_1_ with room temperature unit cell parameters a = 64.5 Å, b = 30.9 Å, and c = 34.8 Å, β = 105.8°. The short diagonal d of the parallelepiped spanned by a and c is 64.4 Å: essentially the length of a. Also, the angle between the diagonal d and cell constant c is 105.6°, which is essentially β. Accordingly, when indexing, either the true cell constant or the diagonal will be randomly assigned to be cell constant a. As should be obvious, the intensities of reflections with like indices in the same pattern indexed with either one or the other indexing convention do not match. This twofold indexing ambiguity can be resolved by correlating intensities of reflections with like indices in two patterns and altering the indices in one of the patterns when the correlation is low [55]. For the above example of myoglobin, the operator h + l, −k, −l needs to be applied (see also [6]). It should be mentioned that the indexing ambiguity for myoglobin cannot result in a merohedrically twinned macroscopic crystal. For this to occur, the ambiguous cell axes must be exact. An example is space group P6_3_, where axes a and b have exactly the same length [4]. There exists the indexing ambiguity h,k,l and k,h,l (minus signs are not necessary in P6_3_ due to symmetry). Under these conditions, (merohedrally) twinned macroscopic crystals might also exist [56]. In other, hopefully rare cases, the inability to distinguish cell axes with almost the same lengths might generate severe problems, especially at low resolution, with highly mosaic crystals containing big macromolecular complexes. Software to process SFX data therefore needs to provide a reliable mechanism to solve the indexing ambiguity (see [55] and http://www.desy.de/~twhite/crystfel/manual-ambigator.html).

### 5.2. Difference Maps

When the indexing ambiguity is solved and the partial intensities are scaled and merged, the integrated intensities can be converted to amplitudes with standard methods [57]. A reference dataset, *F*(*ref*), and a time-resolved dataset, *F*(*t*), are obtained this way. It is imperative to scale the time-resolved amplitudes as accurately as possible to the dark (reference) amplitudes. The program ‘scaleit’ available within the CCP4 suite of programs [57] is particularly useful [4]. Difference amplitudes ΔF=F(t)−F(ref) are then determined. If the unit cell parameters do not change, isomorphous difference maps are calculated from the Δ*F* and phases from the reference model. Atomic displacements caused by structural changes are identified by pairs of positive and negative difference electron density features. Valid negative features are always on top of atoms of the reference structure. Chemically meaningful positive features must be interpreted with a new structure. To find this (unknown) structure can be difficult, since positive difference electron density features and negative features tend to cancel out. In addition, population transfer from the resting state into the reaction is usually quite small: on the order of 5–20% [58]. Especially when the structural changes are small, the void between the features is confusing, and the atoms tend to be shifted too much towards the positive features. Fortunately, there exists a way to produce a conventional map where the population transfer is extrapolated to 100% [59]. A factor *N* of the measured difference, Δ*F*(*obs*), is added to calculated structure factors, *F*(*calc*), determined from the reference state model to obtain extrapolated structure factors, F(ext)=N·ΔF(obs)+F(calc), from which a conventional map can be calculated. When a larger fraction of molecules has been activated in the crystal, *N* is small: on the order of 2–5 [4]. Even when the fractional concentration of activated molecules (FCM) is small, this method is successful. Then, however, *N* is large [60]. The resulting extrapolated map is noisy due to errors caused by the difference Fourier approximation [61] and due to experimental noise in the (then) small difference amplitudes. *N* can be estimated (N_est_) even in the presence of low FCM by subsequently adding more of the Δ*F* to the dark structure factors and inspecting the resulting extrapolated map at positions where there is strong negative difference density in the difference map. This can be conveniently done by carving out a spherical volume from the extrapolated map positioned at locations that display strong difference electron density. The negative (extrapolated) density found within the spherical volume is summed up below a threshold [43,60], and the result (Ʃ_s_) is plotted as a function of N. The appropriate N_est_ is found at the point where Ʃ_s_ starts to diverge [62]. Molecular model(s) (M_int_) can be found which interpret the extrapolated electron density. M_int_ is fit into the extrapolated map by a real space refinement in an appropriate program, such as coot [63], and conventionally refined against the extrapolated amplitudes. In the case of high N_est_ values, quality factors (R_cryst_/R_free_) obtained after the refinement are poor. Nevertheless, a valid M_int_ is obtained that can be tested against the observed difference data. For this, structure factors are calculated, each from the refined M_int_ and the reference structure. From these, difference structure factors are obtained from which a calculated difference map is computed. The calculated difference map can then be compared to the observed difference map. The agreement between the observed and calculated difference maps is inspected, for example, by using an appropriate correlation coefficient, and may be optimized by repeating the analysis with slightly varying factors N_est_ and/or different models. Once the best M_int_ is found, phases for the ΔF are available [43,60] and phased extrapolated structure factors can be determined. A refinement against the phased extrapolated amplitudes usually results in acceptable R factors [4,5,43,58,60]. However, the quality of M_int_ should be primarily based on the similarity (the correlation) between the calculated difference map and the observed difference map. Ideally, M_int_ explains all difference features, and even spurious (positive) difference electron density values are interpreted correctly. This is even more important when the FCM is small and, correspondingly, N_est_ is large.

When the unit cell parameter changes substantially during the reaction, isomorphous difference maps cannot be calculated. Then, ‘omit’ difference maps must be calculated using data obtained after reaction initiation only (see, e.g., [64]). For this, a structural model with the region of interest removed (omitted) is refined against the light data, preferentially with simulated annealing to minimize phase bias. Structure factors *F*(*omit*) are calculated from the model. The omit difference map is then calculated from the amplitudes ΔF=F(t)−F(omit), and phases from the refined (*omit*) model. Extrapolated structure factors, *F*(*ext*), as well as extrapolated maps to guide structural interpretation, may also be calculated if needed: F(ext)=F(omit)+N·[F(t)−F(omit)].

### 5.3. Analysis of Time Series

The ultimate goal of time-resolved crystallography is the determination of the dynamics of the crystalline ensemble from the beginning to the end of a reaction. Chemical kinetics is governed by rate coefficients and chemical kinetic mechanisms that need to be extracted from a comprehensive time series of X-ray data. Approaches based on a component analysis, specifically on the singular value decomposition (SVD), have been developed [42] and successfully applied to synchrotron [58,65,66,67,68,69] and XFEL [5] data. Most importantly, mixtures that inevitably occur during chemical kinetics are separated into pure constituents. For more details, the reader is referred to specialized reviews [43,44,70]. The SVD-based analysis then provides a comprehensive view of the reaction. A compatible chemical kinetic mechanism, the rate coefficients of this mechanism, and the time-dependent concentrations of the intermediate states as well as the time-independent structures of the intermediate states that are populated during the reaction [43] can be determined. Even barriers of activation may be extracted purely from the time-resolved X-ray data [58]. On ultrafast time scales, the description according to chemical kinetics breaks down. Rather than driven by the heat bath, the dynamics is governed by electrostatic interactions on the excited- and ground-state potential energy surfaces (see below). The crystalline ensemble moves in synchrony, since the individual molecules do not have the time to kinetically dephase. Protein quakes [71,72] and collective motions [5,6] become observable. Closely spaced time delays are required on these time scales, and new analysis methods must be developed [73].

## 6. The Blue-Light Receptor Photoactive Yellow Protein

**Figure 2:**
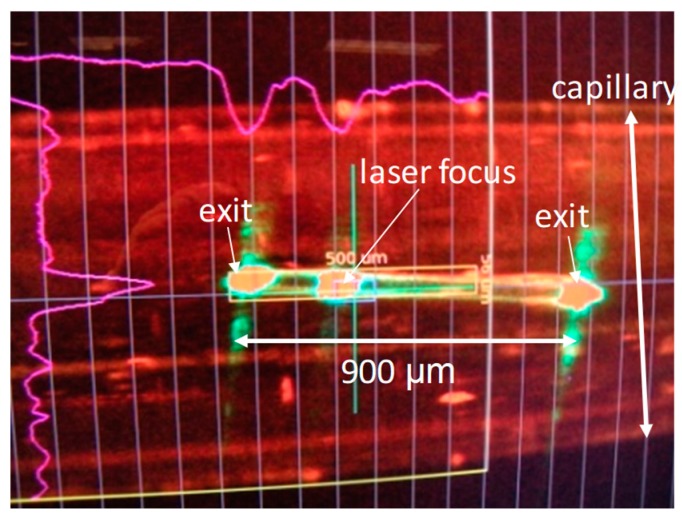
Screenshot of a photoactive yellow protein (PYP) crystal (pale yellow) displayed on a monitor at the XPP instrument of the LCLS during laser illumination. The crystal of size 900 × 40 × 40 µm^3^ is kept in a glass capillary. It is illuminated by a femtosecond laser pulse (laser focus). The crystal apparently acts as a waveguide, with the laser light exiting at both ends.

**Figure 3:**
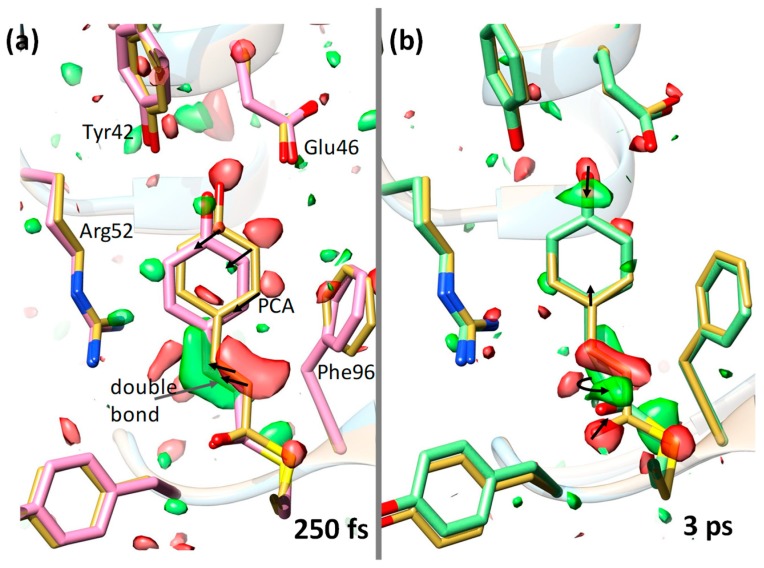
Ultrafast structural changes in the chromophore pocket of PYP [5] Green represents a positive difference electron density and red a negative difference electron density (on the 3/-3 σ contour level). Yellow structure represents a reference (dark state) structure. The p-coumaric acid (PCA) chromophore as well as some nearby residues are marked. (**a**) 250 fs after laser excitation (pink structure); the chromophore configuration is still *trans*. Larger structural changes are denoted by arrows. (**b**) 3 ps after laser excitation (green structure); the structure is *cis*. Isomerization occurred about the double bond (curved arrow) at the chromophore tail. Some structural changes are also shown by arrows.

Ever since its discovery [74], the photoactive yellow protein (PYP) was used to motivate innovations in TRX. Numerous publications in high-ranking journals testify to the importance of this small protein [4,5,59,67,75,76,77]. By absorbing at a wavelength of 460 nm (in the blue region), it senses blue light and it is thought to be responsible for the negative phototaxis [78] of the bacterium (*Halorhodospira halophila*) in which it was originally identified. The central chromophore in PYP is photoactive. It isomerizes from *trans* to *cis* configuration [79], which initiates conformational changes of the protein. After the chromophore isomerization, PYP transitions through a photocycle with several intermediates that acquire subsequent global, but distinct, conformations. Synchrotron-based TR crystallography elucidated the photocycle to almost completion [58,65,67,69,77]. However, the *trans*-to-*cis* photoreaction remained obscure because it is much faster than the pulse duration of X-ray pulses available at synchrotrons. As one of the earliest experiments at the LCLS, an experiment to probe this initial reaction of the PYP photocycle was conducted with macroscopically large (40-µm diameter, ~1-mm long) crystals (Figure 2). This experiment did not produce datasets of sufficient quality to obtain a time-resolved signal [80]. An international consortium of researchers tackled the problem successfully with microcrystals at the CXI instrument of the LCLS [4]. They produced, for the first time for any biological macromolecule, a time-resolved difference electron density (DED) map at the XFEL. The quality of the DED was unprecedented and fully unexpected. At a 1-µs time delay, the DED map showed difference features on the order of 20 times above the noise, which had never observed for PYP at synchrotrons. This experiment then opened the door wide to advance to the femtosecond time range. An experiment with fs time resolution was conducted again at the CXI instrument; this time, however, with the reaction initiated by a femtosecond laser [5]. For the first time, the chemically all- important *trans*-to-*cis* isomerization was seen in real time by observing how electron density clouds shift (Figure 3). Quantum mechanical molecular dynamics simulations show that after light absorption, the PYP is lifted to the electronic excited-state potential energy surface (ES-PES). However, the chromophore configuration is still *trans* on the ES-PES (Figure 3a). The experiment showed, for the first time, macromolecular relaxations on an ES-PES. All other TR-crystallographic experiments to this date featured PYP on the electronic ground state (GS)-PES, where the reaction is driven by the thermal bath. The *trans*-to-*cis* isomerization happens at about 600 fs at the seam between the ES-PES and the GS-PES: the so-called conical intersection. The transition through the conical intersection, as well as the initial relaxation on the GS-PES, was also time-resolved in this experiment. In contrast to conventional chemical kinetics, which is governed by rate coefficients and temperature, the ultrafast relaxations on ES-PES and GS-PES are likely not temperature-dependent. The relaxations are driven by electronic interactions and related structural relaxations after instantaneously accessing the ES-PES. After the transition through the conical intersection, electronic relaxations commence on the GS-PES. The initial events are governed by extreme acceleration of chromophore atoms on the order of 10^14^ m/s^2^. Some of the chromophore atoms reach the speed of sound after 100 fs and collide with the rigid (on this time scale) protein matrix [5]. Most likely, there exist functional modes in the protein which might be excited by this collision. They may facilitate a swift rotation about the chromophore tail’s double bond (Figure 3a) and enhance population transfer into the photocycle. The characteristic time of the relaxation through the conical intersection (~600 fs) and the relatively high primary yield (20%) for photocycle excitation [81] are testimony for this. Eight hundred femtoseconds after excitation, the chromophore is in a very twisted near-*cis* configuration, which relaxes further. The structure at the 3-ps time delay (Figure 3b) is already very similar to the synchrotron-derived structures 100 ps after photon absorption [67,77]. The PYP will continue to drive new experiments and support new ideas. For example, laser pulse shaping techniques [81,82] might be able to control molecules on the ES-PES and steer the transition into the photocycle. High-repetition-rate XFELs are required to collect the necessary X-ray data, which should cover closely spaced time delays in high-dimensional parameter space. New attosecond-pulsed X-ray sources will explore, time-resolve, and remove the notion of instantaneousness from the chromophore excitation itself.

## 7. Transition Metal-Containing Proteins

**Figure 4:**
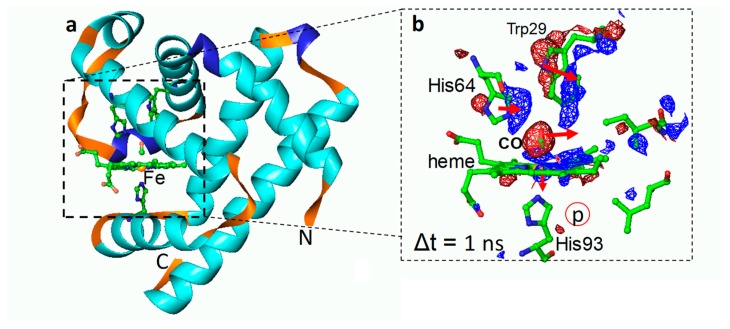
Time-resolved crystallographic photoflash experiment on the L29W mutant of Mb–CO [66]. (**a**) Overall structure of Mb^L29W^–CO in the dark. Dashed box: heme pocket. Some important residues are displayed. The heme iron is shown as a yellow sphere. (**b**) Close-up of the heme pocket 1 ns after an intense optical laser flash to start photodissociation of the CO from the heme. t. The heme and important residues are marked. Red: negative difference electron density; blue: positive difference electron density (−/+ 3 σ contour levels, respectively). Red arrows show structural relaxations at this time delay. In this mutant, the Trp29 transiently occludes the primary docking site of the CO. CO is found at time delays >1 μs on the proximal side of the heme (red-circled p).

Myoglobin (Mb) is a paradigm of a biological macromolecule for biophysicists. It is sufficiently complex, yet small enough to reveal important properties of proteins under controlled conditions. Mb contains a central pigment called heme, which is a protoporphyrin (IX) molecule that coordinates to a single iron atom (Figure 4). If carbon monoxide (CO) is added to Mb, it binds to the iron, forming Mb–CO. Once the CO is flushed away from Mb, early protein relaxations and those related to subsequent geminate rebinding can be observed with time-resolved methods. Ligand migration and protein conformational substates were all discovered with the help of this molecule [66,83,84,85,86,87,88]. Figure 4 shows results from an earlier TR crystallographic study on the L29W mutant of Mb [66]. Already within 1 ns after the CO is flashed away, substantial protein relaxation has occurred, the distal histidine has swung into the heme plane, the CO has migrated away from the heme, and the heme itself is substantially domed with the heme-iron displaced out of the heme plane. The earliest phases of this reaction were then investigated on femtosecond time scales at the XFEL [6]. The trajectory of the CO to its initial docking site could be revealed, as well as initial protein relaxations, extending and confirming earlier results from synchrotron-based TR Laue crystallography [66,85,87,88] and providing a solid structural base for ultrafast spectroscopic findings [89,90].

Transition metals are ubiquitous in biology. Iron is probably most abundant, but other metals such as copper and manganese are also found in important proteins and enzymes. Examples include the heme-containing cytochrome-c nitrite reductase [91,92], the manganese- and iron-containing photosystem II [33,93,94], the terminal oxidase in the respiratory chain (copper and iron) [95], as well as various superoxide dismutases (with iron, manganese, copper, or other metals in their active sites) [96,97]. There are many more metalloproteins [98]. Most of them catalyze reactions of biologically and biomedically high importance, which merits investigation of their catalytic mechanism. Care has to be taken that the transition metal is not photoreduced during X-ray structure analysis [99]. Otherwise, the change of the oxidation state might lead to rapid structural changes that may be significant, even during the ultrafast fs X-ray pulses at XFELs [19,100]. Although the diffraction-before-destruction principle [17] suggests that atomic displacements caused by radiation damage can be neglected, there exist cases, such as in photosystem II, where rapid configurational changes could affect the interpretation of catalytic mechanisms [33,101,102,103]. In other cases, where conformational changes are large, small effects due to radiation damage might play a smaller role. In TR-SFX, the crystal is discarded after each X-ray pulse and the next crystal is new and pristine. As a result, a reaction initiated in these crystals is completely unaffected by radiation damage until it is probed. This is in contrast to the synchrotron, where radiation damage of a crystal by multiple X-ray pulses (or by a longer X-ray pulse) might also impair the kinetics [62]. However, also at the synchrotron, the feasibility of serial macromolecular crystallography has been shown with monochromatic and pink beams [47,104,105,106]. Such an approach would also provide a clean, essentially X-ray-damage-free description of the progress of a macromolecular reaction. Still, the initiation of reactions in crystals, especially in enzyme crystals, remains a challenge. Methods to start reactions by laser pulses are fairly established, but most proteins and especially enzymes are not light-sensitive. Even if they contain a light-sensitive cofactor, its illumination with light would likely not be functional and would not trigger an enzymatic reaction. Apart from inactive caged substrates that could be soaked into enzyme crystals and activated by laser pulses [107,108,109], there are only a few examples where light absorption actually may be used to control enzymatic activity. Some such examples are mentioned below. Other examples include enzymes that are engineered to be coupled to light-sensitive domains or moieties whose light activation would change the enzymatic activity [110,111].

## 8. Enzymes

Enzymes are biocatalysts; they perpetuate the catalytic functions of life, and thus, they must work properly and their functions must be regulated. If their functions are compromised, severe diseases may result. Enzymatic reactions decide the fate of an organism. Cancer, for example, often results from out-of-control enzymatic function, as comprehensively described in textbooks [112]. On the other hand, pathogens causing infectious diseases can be destroyed by targeting their essential enzymes. Hence, it is of utmost importance to investigate enzymatic activity, understand catalytic mechanisms, and explore opportunities to control their function. The “holy grail” of TRX is therefore the ability to investigate reactions and interactions of enzymes with their specific substrates in real time. At synchrotrons, these experiments are complex because a number of X-ray exposures (detector readouts) from a single macroscopic crystal are usually required to produce a complete dataset. This may require flow cells or other means [113,114] to load the crystal with substrate and remove product after each exposure. To initiate the enzymatic reaction in the crystals, inactive, caged substrates may be required that first need to diffuse into the crystals, and are subsequently activated by laser pulses [107]. Crystals of many proteins, especially biomedically important ones, tend to scatter weakly and exhibit large mosaicities. Both may impair the quality of the (monochromatic and especially Laue) diffraction patterns collected at synchrotrons. Despite these demanding requirements, a number of studies have been successfully conducted [3,16,115,116]. However, a transformative step forward would be the elimination of experimental difficulties to the point such that routine investigations become generally feasible on all sorts of biomacromolecular reactions. Serial crystallography with microcrystals offers a practicable approach. Since the crystals are so small, they can be mixed with substrate quickly and exposed one-by-one to the X-ray beam at a time Δt after mixing. No flow cells are required this way. A successful application of this approach has been demonstrated recently [64] and is described below.

**Figure 5:**
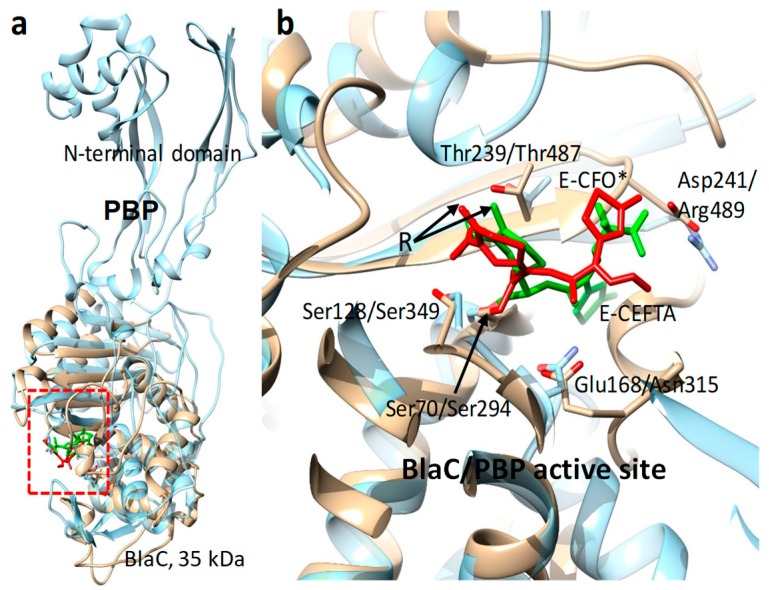
Structural comparison of penicillin-binding protein (PBP, blue, PDB entry 3OCN) and *M. tuberculosis* β-lactamase (BlaC; brown, PDB entry 6B69). (**a**) Overall view: BlaC is structurally very similar to the C-terminal domain of the PBP. (**b**) Detailed view of the active sites with essential amino acids in BlaC/PBP shown according to numbering convention. The cephalosporin antibiotics ceftriaxone (CFO*, red) and ceftazidime (CEFTA, green) are bound to serine residues (arrow) in the active sites of BlaC and PBP, respectively. The leaving group of the cephalosporins (R, arrows) is cleaved off.

The first enzyme structure to be solved was that of lysozyme in 1965 [117]. Lysozyme catalyzes the hydrolytic separation of 1,4-beta-links between N-acetylmuramic acid and N-acetyl-D-glucosamine of bacterial cell walls; hence, it has antimicrobial activity. In chicken egg whites and human nose mucus, lysozyme is the first line of defense against bacterial infections. Ever since, researchers have been wondering how this enzyme performs its catalytic function. The static structure reveals clues, such as the structure of the catalytic cleft and the positions of catalytically important amino acid residues. However, as the X-ray structure is static, the precise catalytic mechanism is difficult to determine. Even more important than lysozyme in the fight against infectious diseases are the so-called penicillin-binding proteins (Figure 5a). These enzymes catalyze the linkage of N-acetylmuramic acid to *N*-acetyl-d-glucosamine to form the cell wall in the bacteria, a reaction just opposite to the lysozyme reaction. β-lactam antibiotics such as penicillins and chemically similar compounds such as the cephalosporins irreversibly inhibit the penicillin-binding protein [118]. Once blocked by these compounds, the enzyme is not able to maintain the integrity of the cell wall and the bacteria perish. Unfortunately, resistance against antibiotics is rampant. β-lactamases are found among a disturbingly large number of possible resistance mechanisms [119,120,121]. These enzymes modify penicillin and related compounds by opening the peculiar, relatively unstable β-lactam ring of these compounds. Some β-lactamases are strikingly similar to the catalytic domain of the penicillin-binding proteins (Figure 5a). Even the catalytic clefts are essentially identical (Figure 5b). In contrast to the penicillin-binding protein, which is irreversibly and covalently modified by the antibiotic, the β-lactamases bind the antibiotic, catalyze the ring opening, and are finally able to hydrolyze and release the modified product. The free enzyme can now engage in a subsequent cycle of antibiotic modification, eventually rendering the entire amount of administered antibiotics ineffective. The catalytic cycle of a typical β-lactamase lasts on the order of 1 s, with several intermediates persisting on the millisecond time scale [122]. The enzyme substrate complex forms within ten ms, after which an active-site amino acid residue attacks the lactam ring, opens it, and covalently binds the reaction product. The *Mycobacterium tuberculosis* β-lactamase (BlaC), an Ambler class-A β-lactamase [123], has been investigated recently at the XFEL [64,124]. Experiments on this β-lactamase was driven by three important considerations: (i) BlaC is biologically and biomedically highly significant. Hence, results are widely important. This justifies the use of limited beamtime at the XFEL. (ii) The reaction catalyzed by BlaC is simple enough that the enzyme can be used as a model system to establish structure-based enzymology at the XFEL. (iii) BlaC forms well-diffracting microcrystals, and β-lactam antibiotics are readily available and very soluble in most cases. The approach to investigate the BlaC reaction has been named ‘mix-and-inject’ serial crystallography (MISC) [124]. In MISC, micron-sized enzyme crystals are mixed with substrate before the mixture is injected into the X-ray interaction volume. The substrate diffuses into the crystals and initiates a reaction. Since the crystals are so small, diffusion times are fast; much shorter than the time required for one catalytic turnover. If the BlaC reaction can be followed, this approach will also work with other important enzymes.

Diffusion is based on Fick’s laws, the second of which is:

(1) $$D\nabla^{2}C=\frac{\partial }{\partial t}C$$

where *D* is the diffusion coefficient, ∇^2^ is the Laplace operator, and *C* is the time- and space-dependent concentration of substrate in the crystals, which is initially zero. This partial, second-order differential equation can be solved subject to certain boundary conditions [125]. Diffusion times to the center of the crystal can be estimated this way. They approach 1 ms for crystals of about 1–5 µm edge lengths, depending on the substrate. In addition, Fick’s first law relates the flux ($\vec{J}$, the velocity with which the substrate enters the crystal) to the concentration gradient: $\vec{J}=-D\vec{\nabla }C$, with $\vec{\nabla }$ being the ‘nabla’ operator. Hence, the larger the substrate concentration outside the crystal, the higher the flux into the crystal, and the faster appropriate concentrations accumulate in the crystals. To reach proper occupancy of the enzyme–substrate complex, the number of substrate molecules should at least match the number of catalytic centers in the unit cell. This is called stoichiometric concentration. According to Fick’s first law (and also evident from the solutions to Fick’s second law), given properly high initial outside substrate concentrations, stoichiometric concentrations may be reached about an order of magnitude faster than the characteristic diffusion time, even faster than the mixing time (Table 1). Accordingly, the experiment’s best time resolution is reached if the substrate is mixed with enzyme microcrystals rapidly, the initial substrate concentration is high, and crystals are as small as possible. Sub-ms time resolution is possible. These requirements led to the development of optimized ‘mix-and-inject’ injector devices [64,126], where mixing is achieved efficiently to reach the required time resolution. Table 1 lists relevant parameters for a 30-ms time delay after mixing BlaC microcrystals with the cephalosporin antibiotic ceftriaxone (CEF) as substrate. Mixing plus characteristic diffusion times are on the order of 8 ms, and stoichiometric concentration is reached even within the mixing time (5 ms). This is more than sufficient to time-resolve the initial 30-ms time delay.

**Figure 6:**
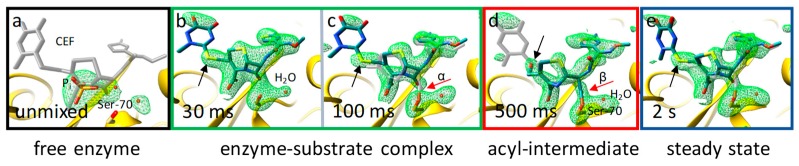
Active site structures during the BlaC reaction with CEF [64] as determined by MISC. Simulated annealing ‘omit’ difference density shown in green on the 2.5 σ level. (**a**) Catalytic cleft of the free enzyme, phosphate (P_i_), and water is present. Ser-70 is marked. The position of CEF is shown in gray as a guide to the eye. (**b**) Catalytic cleft 30 ms after mixing; resolution 2.75 Å. Strong electron density shows the CEF ligand. (**c**) Catalytic cleft 100 ms after mixing; resolution 2.15 Å. Gap in electron density (red arrow, α) shows that the covalent bond has not yet formed. Black arrows in (**b**,**c**) point to the sulfur of the leaving group. (**d**) Catalytic cleft 500 ms after mixing; resolution 2.2 Å. The gap between the CEF and Ser-70 is closed, indicating a covalent bond (red arrow, β). The sulfur feature (black arrow, behind the hydroxyl) is gone. (**e**) Catalytic cleft in the steady state. Similarities to the 30-ms and 100-ms time delays are evident.

The catalytic reaction of the *M. tuberculosis* BlaC with CEF has been observed from 30 ms to 2 s with MISC experiments performed at the LCLS [64,124]. CEF is already fully occupied in the catalytic cleft at 30 ms (Figure 6b). Next, the Ser-70 residue of the enzyme attacks the β-lactam ring (Figure 6c,d). This nucleophilic attack does not only open the ring, but also leads to the simultaneous release of a leaving group (R) from CEF. Very little evidence of this process is present at both 30 ms and 100 ms. However, at 500 ms, a covalent bond between CEF and Ser-70 has formed (Figure 6d). Since the leaving group’s electron density is not well resolved due to high mobility, its ring electron density is a poor indicator of its presence (or release). The missing density on the sulfur atom of the leaving group, however, indicates that this group is split off. At 30 ms to 100 ms, the structure of the enzyme–substrate complex, and at 500 ms, the structure of the acyl complex can be determined. Two seconds after mixing, the situation is not that clear; apparently, the enzyme-substrate (ES) complex reappears (Figure 6e). The electron density looks similar to those for the 30-ms and 100-ms time delay. Since 2 s is a timepoint well in the steady state of the enzyme, one would expect that the most stable state, which should be the acyl complex, dominates the electron density. However, this is not the case, since the ES complex is observed at 2 s. A more quantitative view is obtained when the coupled differential equations of the kinetic mechanism are integrated. The concentration profiles obtained can then be compared to the results from the MISC experiment. A compatible concentration profile can only be obtained when the rate of the nucleophilic attack reaction of Ser-70 onto the β-lactam ring is effectively blocked. Only then does the ES complex accumulate in the steady state. Although product inhibition has been known for cephalosporin substrates [127], this is the first time that such an inhibition has been proposed based on structural evidence, and may be useful for therapeutic applications. Additional experiments with closely spaced time points are necessary to determine the exact mechanism(s) of this inhibition.

## 9. Other Light-Controlled Proteins and Enzymes

Membrane-bound receptors usually obtain catalytic activity when they are activated. The activity is triggered by the binding of an effector, such as a hormone, and transduced through the cell membrane to the cytoplasmic side. More downstream effectors bind to the receptor and are thereby activated. This leads to signal amplification that then causes changes in the cell behavior, such as growth and differentiation [112]. Light is also sensed by membrane-bound receptors which transmit the light signal into the cell. The most well-known of these is certainly the eye pigment rhodopsin [128,129]. Rhodopsins are seven-helix transmembrane proteins found in higher organisms as well as in bacteria, with diverse activities ranging from light perception to cation and anion transport [130]. The bacterial rhodopsin (bacteriorhodopsin, BR) found in halophilic purple bacteria [131] is likely the most investigated of them all. Upon light absorption, it pumps protons out of the bacterium, hence generating a proton gradient across the cell membrane. This proton gradient is used by another enzyme (ATP synthase) to produce the cell fuel ATP (adenosine triphosphate). This makes BR interesting for potential applications using sunlight to generate chemical energy. The central chromophore in BR is an all-*trans* retinal bound to a lysin, forming a general structure called a Schiff base. After absorption of green light (λ_max_ = 520 nm), the retinal undergoes a *trans*-to-*cis* isomerization reaction that drives BR in a photocycle with multiple intermediates [132]. High-quality crystals of BR were obtained using a method developed by Landau and Rosenbush called lipidic cubic phase (LCP) crystallization [133,134,135]. Despite several decades-long successful structure determination studies of BR at synchrotrons, it has been difficult to investigate BR with the Laue method at room temperature. Crystals scatter weakly and are strongly radiation sensitive. This prevented TRX experiments on BR until the XFELs became available. Microcrystals of BR obtained through LCP crystallization can be extruded into the XFEL beam using the viscous matrix injector. Ultrafast time-resolved SFX investigations with the pump–probe technique become possible this way [25,26]. In BR, the Δ13–14 double bond near the Schiff base isomerizes on an ultrafast time scale. The Schiff base carbon moves substantially by about 2 Å to accommodate the isomerization [136]. This then has a profound influence on the conformations of BR intermediates and the water network within the protein, and it provides a structural basis of how the BR performs its proton-pumping function [26,136].

**Figure 7:**
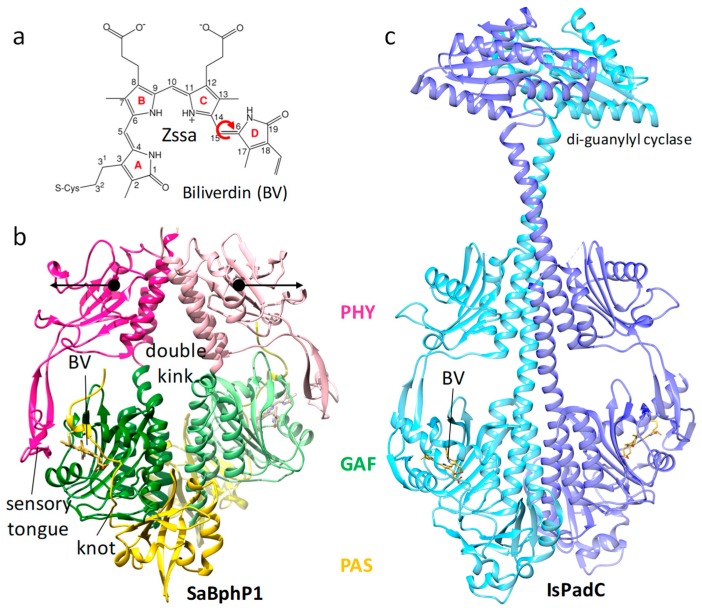
Bacterial phytochrome structures. (**a**) Structure of the central chromophore, biliverdin (BV). In dark-adapted BphPs, biliverdin (BV) is found in most cases in the Z syn–syn–anti configuration. Red arrow: isomerization upon red light absorption. (**b**) Structure of the myxobacterial phytochrome 1 (SaBphP1) photosensory core module; PDB entry 6BAO [32]. PAS, GAF, and PHY domains are colored yellow, green, and magenta, respectively. The sensory tongue, the knot, and the BV are marked. Black arrows: structural displacements of the PHY domains after light absorption; PDB entry 6BAO. (**c**) Structure of the full-length *Idiomarina* spp. phytochrome-activated diguanylyl cyclase (IsPadC), pdb entry 5LLW [142].

Absorption of light can regulate enzymatic activity in soluble proteins. The light is sensed in a photoactive moiety, and the signal is subsequently transferred to a domain with enzymatic activity. Ideally, the light sensor and the enzyme are located on the same protein. Such a photoresponsive enzyme was discovered in plants 80 years ago [137,138] and was called phytochrome. Effects of phytochromes on plant morphology and physiology are numerous. Examples are etiolation, greening, shade avoidance, and leaf expansion [139]. Plant phytochromes are difficult to crystallize, especially in their full-length, enzymatically active, functional form. The discovery of homologous phytochromes (BphPs) in nonphotosynthetic bacteria has been a breakthrough [140,141], since these BphPs tend to crystallize readily. Crystal structures are now known for BphPs of about a dozen bacteria [32,138], including structures of full-length BphPs [142,143] (see also Figure 7c) and a plant phytochrome B [144]. The phytochromes consist of four domains, three of which, called PAS, GAF, and PHY, form the photosensory core module (PCM), and the fourth is an effector domain with enzymatic activity (Figure 7b,c). Red light is absorbed by the central chromophore, which for BphPs is usually biliverdin (BV) (Figure 7a). The chromophore undergoes a configurational change. The Δ15–16 double bond connecting rings C and D isomerizes from the Z-configuration (Figure 7a) to the E-configuration. This signal is picked up by a complex network of amino acid interactions [32,145] and relayed towards the sensory tongue of the PHY domain (marked in Figure 7b). The sensory tongue completely changes its conformation from a β-sheet to an α-helix [146,147]. In response, the PHY domain moves substantially (see arrows in Figure 7b), hence opening the PCM [146,147]. As the effector domain with enzymatic activity is linked to the PHY domain (Figure 7c), it must undergo large structural changes that are caused by the PHY domain displacements. The characterization of the signal transduction through the PCM towards the effector domain is subject to intense investigations. First, the chromophore isomerization must be structurally explored, and second, the signal transduction must be identified in real time by time-resolved experiments. Whether crystals will survive the large structural changes is a matter of debate. However, microcrystals used at XFELs seem to be surprisingly robust towards changes in the unit cell and tolerate even space group changes [148], so chances of being able to observe this important photoreaction are excellent. In addition to the significance of the phytochrome reaction for bacterial behavior and development [32], the identification of key amino acid residues in the mechanism of this isomerization would have biomedical applications. They guide the design of new fluorescent biomarkers in the far red [149] for deep tissue investigations [150], Förster resonance energy transfer (FRET) applications, or optical applications with super-resolution [151].

## 10. Outlook

The investigation of the catalytic functions of many soluble and membrane-bound biologically and biomedically highly significant enzymes with the mix-and-inject technique are at the frontier of macromolecular TR-SFX [64,124,148]. The structural characterization of electron transfer reactions in photosystem I (PSI) and photosystem II (PSII), as well as the water splitting catalyzed by the oxygen-evolving complex (OEC) in PSII, is ongoing, with spectacular results [33,93,103]. Proposals on numerous other light-sensitive proteins and enzymes await receiving beamtimes at the existing XFELs. Ligand binding to and signal transduction by membrane-bound receptors, such as the many G-protein coupled receptors [152] and other biologically highly significant receptors, await investigation. High-repetition-rate XFELs will reduce experimental times and keep protein consumption to a minimum. The way in which experiments will be conducted will become very different from today. Hundreds or even thousands of different microcrystalline samples can be examined in a few hours by specialized staff at most sophisticated high-repetition-rate XFELs. Accordingly, hundreds of comprehensive time series may be collected in one shift of XFEL beamtime using different substrates covering a vast parameter space. As a result, crystallo-kinetic information obtained by TR-SFX (or potentially time-resolved serial crystallography at synchrotrons) will become a potent tool in the fight against cancer and infectious diseases.

## Figures and Tables

**Table 1:** Time scales and other parameters important for consideration in a mix-and-inject experiment aimed to investigate an enzymatic reaction at a time delay of 30 ms after mixing.

Delay	Mixing Time	Crystal Size (μm^3^)	Diffusion Coefficient (cm^2^·s^−1^)	Diffusion Time	Substrate after Mixing	Time to Reach Stoichiometric Concentration
30 ms	5 ms	10 × 10 × 3	2.3 × 10^−6^	3.5 ms	240 mmol/L	0.24 ms
